# Supplementary material for: Palladium concave nanocrystals with high-index facets accelerate ascorbate oxidation in cancer treatment
Source: Nat Commun. 2018 Nov 19;9:4861. doi: 10.1038/s41467-018-07257-z (PMC6243004; doi:10.1038/s41467-018-07257-z)
Supplement: Supplementary file 1 — Supplementary Information [file 41467_2018_7257_MOESM1_ESM.doc]

**Supplementary Information**

**Palladium Concave Nanocrystals with High-index Facets Accelerate Ascorbate Oxidation in Cancer Treatment**

**Chong *et al.***

**Supplementary Figure 1. Size distribution of Pd nanocrystals measured by TEM.** (a) Pd nanocubes (Pd NCs). (b) Pd concave nanocubes (Pd CNCs).

**Supplementary Figure 2. TMB oxidation catalyzed by Pd nanocrystals.** (a) Evolution of UV-vis absorption spectra for 800 μM TMB in the presence of 5 μg/mL Pd nanotubes (Pd NCs) or Pd concave nanotubes (Pd CNCs) at room temperature after 30 min incubation. (b) Time-dependent absorbance changes of TMB oxidation products at 652 nm with different concentrations of Pd CNCs. (c) The temperature-dependence of the catalytic activity of Pd CNCs for TMB oxidation. (d) The pH-dependence of the catalytic activity of Pd CNCs for TMB oxidation. Data are expressed as the mean ± standard deviation. Error bars were taken from three parallel experiments (n = 3).

**Supplementary Figure 3. Ion scan mass spectra of ascorbic acid oxidation.** (a) [M-H]^-^ of ascorbic acid (formula: C_6_H_8_O_6_). (b) [M-H]^-^ of dehydroascorbate (DHA) (formula: C_6_H_6_O_6_). (c) [M-H]^-^ of ascorbic acid catalyzed by Pd concave nanocubes.

**Supplementary Figure 4. The dissociative adsorption of O_2_ on the Pd {111} facet.** (a) Top view of DFT optimized structures of O_2_ molecule adsorbed by Pd{111} surface. (b) The electrostatic potential of the electron density distributed on Van der Waals surfaces (isodensity=0.001 a.u.) of O_2_@ Pd{111}. The green arrows in (b) point to the global EPS minima of the electron density VDW surface.

**Supplementary Figure 5. Effects of ascorbate on cancer and normal cells.** Cell viability assay in the presence of ascorbate for 24 h after HCT116 cells (a) and IEC6 cells (b) were plated at the same density. The concentration of ascorbate varies from 0 to 20 mM. The data are expressed as the mean ± standard deviation. Error bars were taken from three parallel experiments (n = 3).

**Supplementary Figure 6. Effect of Pd concave nanocubes (Pd CNCs) on the ascorbate induced cytotoxicity.** Cell viability of HCT116 cells (a) and ICE6 cells (b) treated with 2.5 mM ascorbate in combination with different concentration of Pd CNCs varying from 0 to 100 μg/mL. The data are expressed as the mean ± standard deviation. Error bars were taken from three parallel experiments (n = 3).

**Supplementary Figure 7.** **Effects of Pd concave nanocubes (Pd CNCs) on cell viability.** The viability of HCT116 cells (a) and IEC6 cells (b) treated with 0-100 μg/mL Pd CNCs for 24 h. The data are expressed as the mean ± standard deviation. Error bars were taken from three parallel experiments (n = 3).

**Supplementary Figure 8. PARP activation in HCT116 cells.** Immunoblots for PRAP and β-actin from cell lysates after 2 mM ascorbate or 50 μg/mL Pd CNCs treatment.

**Supplementary Figure 9. Biodistribution and excretion of Pd concave nanocubes (Pd CNCs).** (a) Time-dependent biodistribution of Pd CNCs in female Balb/c mice. (b) Pd levels in urine and feces after injection. Injection dosage: 0.1 mg Pd CNCs. The numerical data are expressed as the mean ± standard deviation. Error bars were taken from three parallel experiments (n = 3).
